# *nab*-Paclitaxel for the treatment of breast cancer: an update across treatment settings

**DOI:** 10.1186/s40164-017-0066-5

**Published:** 2017-03-22

**Authors:** Adam Brufsky

**Affiliations:** Division of Hematology/Oncology, University of Pittsburgh, 300 Halket Street, Suite 4628, Pittsburgh, PA 15213 USA

**Keywords:** Breast cancer, Immunotherapy, *nab*-Paclitaxel, Pathological complete response

## Abstract

**Purpose:**

The purpose of this systematic review is to discuss recent studies and ongoing trials of *nab*-paclitaxel in breast cancer and to examine the potential role of *nab*-paclitaxel as a backbone for immuno-oncology therapies.

**Methods:**

PubMed and selected congress proceedings were searched for studies of *nab*-paclitaxel in breast cancer published between 2013 and 2015. All phase II and III clinical trials, retrospective analyses, and institutional studies were included. Active, ongoing, phase II or III trials on *nab*-paclitaxel that were listed on ClinicalTrials.gov were also included.

**Results:**

Sixty-three studies, including 23 in early-stage and 30 in metastatic breast cancer (some studies not classifiable by setting), were included in this analysis. Trials of neoadjuvant *nab*-paclitaxel–containing regimens have reported pathological complete response rates ranging from 5.7 to 53%. Median overall survival in metastatic breast cancer studies ranged from 10.8 to 23.5 months, depending on dose and regimen. Adverse event profiles of *nab*-paclitaxel were generally similar to those reported from previous studies. Several ongoing trials are evaluating *nab*-paclitaxel in the early-stage and metastatic settings, including in combination with immuno-oncology agents.

**Conclusions:**

*nab*-Paclitaxel continues to demonstrate promising efficacy in breast cancer. Recent studies demonstrate high pathological complete response rates in early-stage breast cancer, particularly in triple-negative breast cancer, an area of high unmet need, and encouraging overall survival in metastatic breast cancer across doses and schedules. Ongoing trials will provide further insights into the role of *nab*-paclitaxel in breast cancer including use as a potential backbone chemotherapy agent for immuno-oncology therapies such as checkpoint inhibitors.

## Background

Breast cancer remains the most commonly diagnosed cancer among women in the United States and worldwide [https://www.cancer.org/content/dam/cancer-org/research/cancer-facts-and-statistics/annual-cancer-facts-and-figures/2017/cancer-facts-and-figures-2017.pdf, http://www.cancer.org/acs/groups/content/@research/documents/document/acspc-044738.pdf]. Globocan estimated that 1.7 million new cases of breast cancer were diagnosed and that more than half a million women died from breast cancer in 2012 [http://www.cancer.org/acs/groups/content/@research/documents/document/acspc-044738.pdf]. The majority of patients (61%) present with localized disease [http://seer.cancer.gov/statfacts/html/breast.html]. Regional disease is diagnosed in 32% of patients, and 6% present with distant metastatic disease [http://seer.cancer.gov/statfacts/html/breast.html]. The overall 5-year survival for all stages combined is 89% [http://seer.cancer.gov/statfacts/html/breast.html]. However, survival rates vary by stage. Localized disease is associated with a 5-year survival rate of 99%, regional disease is associated with a 5-year survival rate of 85%, and metastatic disease is associated with a 5-year survival rate of 26% [http://seer.cancer.gov/statfacts/html/breast.html].

Treatments for localized breast cancer consist of surgical resection with or without radiation therapy [1]. Neoadjuvant chemotherapy is recommended for large tumors (stage IIA-B or T3N1M0). The primary approach for managing metastatic breast cancer (MBC) is systemic therapy, consisting of cytotoxic chemotherapy, endocrine therapy for hormone receptor–positive disease, and HER2-targeted agents for HER2-positive cancers. Many of the neoadjuvant, adjuvant, and metastatic chemotherapy regimens preferred by the National Comprehensive Cancer Network include paclitaxel [1]. One disadvantage of paclitaxel is the development of hypersensitivity reactions to the solvent, Kolliphor EL (formerly called Cremophor EL) [2]. Nanoparticle albumin-bound paclitaxel (*nab* ^®^-paclitaxel, Celgene Corporation, Summit, NJ) is solvent-free, minimizing hypersensitivity reactions and potentially other solvent-related toxicities, such as neutropenia [3–5]. Due to minimal risk of hypersensitivity, premedication with prophylactic steroids is not required [3, 4]. Another advantage of *nab*-paclitaxel vs standard paclitaxel is the increased rate of transport across endothelial cell layers, greater and faster tissue penetration, and slower elimination of paclitaxel [6–8]. *nab*-Paclitaxel also demonstrates increased intratumoral delivery and retention, resulting in 33% higher intratumoral drug concentrations [6]. Compared with paclitaxel, *nab*-paclitaxel yields a 10-fold higher mean maximal concentration of free paclitaxel [8].

*nab*-Paclitaxel is currently approved for locally advanced or metastatic non-small cell lung cancer, metastatic pancreatic cancer, and MBC that has progressed on combination chemotherapy or relapsed within 6 months of adjuvant chemotherapy [2]. The approval in MBC was based on a randomized phase III trial of *nab*-paclitaxel 260 mg/m^2^ vs paclitaxel 175 mg/m^2^ every 3 weeks (q3w). *nab*-Paclitaxel demonstrated a significantly higher overall response rate (ORR; 33 vs 19%; *P* = 0.001) and longer time to tumor progression (23 vs 17 weeks; hazard ratio [HR], 0.75; *P* = 0.006) vs paclitaxel in the intention-to-treat (ITT) population [5]. Overall survival (OS) was not significantly different between the 2 treatment groups in the ITT population; however, in the second- or later-line setting, OS was significantly longer for *nab*-paclitaxel vs paclitaxel (median, 56 vs 47 weeks; HR, 0.73; *P* = 0.024). Significantly less grade 4 neutropenia (9 vs 22%; *P* < 0.001) and more grade 3 sensory neuropathy (10 vs 2%; *P* < 0.001) were reported with *nab*-paclitaxel [5]. Grade 3 sensory neuropathy in patients who received *nab*-paclitaxel improved to a lower grade after a median of 22 days of treatment interruption.

Recent studies have examined *nab*-paclitaxel in early-stage breast cancer, primarily as a neoadjuvant agent. Studies also continue to evaluate the efficacy of *nab*-paclitaxel in MBC in combination and across doses and schedules. This review summarizes data from recent studies of *nab*-paclitaxel across breast cancer settings, discusses ongoing trials, and provides perspectives on the future role of *nab*-paclitaxel in breast cancer.

## Methods

PRISMA guidelines were followed in this systematic review. PubMed was searched for articles published between January 1, 2013 and February 28, 2016. Abstracts from the American Society for Clinical Oncology (ASCO) annual meeting and the ASCO Breast Cancer Symposium 2013–2015 were included. The entry terms for the search were “*nab*-paclitaxel” and “breast.” Abstracts from the 2014 European Breast Cancer Conference and the 2013–2015 San Antonio Breast Cancer Symposium proceedings were searched using the term “*nab*-paclitaxel.” Phase II and III clinical trials, retrospective analyses, and institutional studies were included. Duplicate studies, topic reviews, case studies, nonhuman or preclinical studies, and non-English articles were excluded. One article in PubMed was embargoed and inaccessible.

## Results

**Fig. 1:**
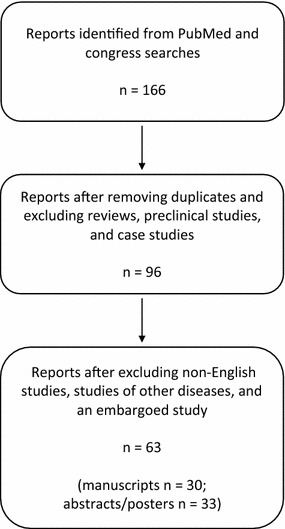
Schematic of literature search

The publication selection process is depicted in Fig. 1. Twenty-three studies of *nab*-paclitaxel in early-stage breast cancer were retrieved, including 21 neoadjuvant and 2 adjuvant studies. Three post hoc analyses of previous neoadjuvant trials were included in the 21 retrieved neoadjuvant studies. Studies of neoadjuvant *nab*-paclitaxel in early-stage breast cancer are presented in Table 1. There were also 30 studies of *nab*-paclitaxel in MBC, including 3 health economic analyses. Studies of *nab*-paclitaxel in MBC that reported progression-free survival (PFS) or OS are presented in Table 2. Only studies with ≥50 patients are detailed in the text; however, all reports on early-stage HER2+ disease were included because of the small number of studies.

**Table 1:** Pathologic complete response in recent neoadjuvant studies of *nab*-paclitaxel in early-stage breast cancer

Study, author, year	Type of study	N (ITT)	Patient population or stage of disease	Regimen	pCR definition	Overall pCR, %	Subgroup pCR, %	*P*
Unselected (all subtypes; n = 7)								
GeparSepto, Untch, 2016 [11]	Phase III	1229	High-risk early-stage BC	*nab*-P 125 (150 before amendment) mg/m^2^ qw	ypT0 ypN0	38.4	TNBC, 48.2%	Overall, <0.001
				Pac 80 mg/m^2^ qw		29.0	TNBC, 26.3%	TNBC, <0.001
Huang, 2015 [9]	Phase II	120	Stage II–III	*nab*-P 125 mg/m^2^ + carbo AUC 2 qw every 21 days (+trastuz if HER2+)	ypT0/is ypN0	*nab*-P, 26.7	NR	0.904
				Pac 80 mg/m^2^ + carbo AUC 2 qw every 21 days (+trastuz if HER2+)		Pac, 25.6		
Shigematsu, 2015 [10]	Phase II	55	Operable T1c3N02M0	*nab*-P 260 mg/m^2^ q3w + cyclophosphamide 600 mg/m^2^ → FEC q3w	Necrosis and/or absence of all tumor cells or replacement of cancer cells with granulation and/or fibrosis in breast and axilla	37	HR+/HER2−: 8 HR+/HER2+: 56 HR−/HER2+: 63 TNBC: 62	NA
Seki, 2015 [57]	Phase II	40	Stage I–III	*nab*-P 80 mg/m^2^ qw 3/4 → FEC q3w	ypT0/is ypN0	40	Luminal A: 20 Luminal B/HER2−: 15.4 Luminal B/HER2+: 60 HER2 enriched: 80 TNBC: 42.9	NA
Neonab, Khasraw, 2015 [58]	Phase II	40	Stage II–III	EC → *nab*-P 125 mg/m^2^ qw 3/4 (+trastuz if HER2+)	NR	NA	NA	NA
Tsugawa, 2014 [59]	Phase II	34	cT1c-3/N0-1/M0 or T1/N1/M0	*nab*-P 150 mg/m^2^ qw 3/4 → FEC q3w	ypT0/is N0	27	ER+: 9 ER−: 58	NA
Khan, 2015 [12]	Phase II	32	Stage II–III operable BC with low HER2 expression	*nab*-P 100 mg/m^2^ + trastuz (4 mg/kg, then 2 mg/kg) qw → ddAC	In breast and axilla	22	In axilla of patients with positive axillary biopsy: 53 In breast of patients with grade 3 tumors: 41	NA
HER2− or TNBC (n = 9)								
SWOG S0800, Nahleh, 2014 [14]	Phase II	215	HER2− IBC or LABC	*nab*-P 100 mg/m^2^ qw + bev → ddAC + PEG-G	ypT0 ypN0	28	With bev: 36	0.021
				*nab*-P 100 mg/m^2^ → ddAC + PEG-G vs AC + PEG-G followed by *nab*-P			No bev: 21	
Kuwayama, 2015 [19]	Phase II	152	Stage II–III HER2−	*nab*-P 100 mg/m^2^ qw 3/4 → FE100C	ypT0/is ypN0	17	TNBC: 30	Overall, 0.323 TNBC, 0.866
				Docetaxel 75 mg/m^2^ q3w → FE100C		12	TNBC: 28	
ADAPT TN, Gluz, 2015 [17, 18]	Phase II	130	TNBC	*nab*-P 125 mg/m^2^ qw 2/3 + carbo AUC 2	ypT0 ypN0	49.2		<0.001006
				*nab*-P 125 mg/m^2^ qw 2/3 + gem 1000 mg/m^2^ qw 2/3		25.0		
GEICAM, Martin, 2014 [13]	Phase II	81	HER2−/ER+	*nab*-P 150 mg/m^2^ qw 3/4	RCB 0 + 1	24.7	ER+: 23.3 HER2−: 23.3	NA
TBCRC 008, Connolly, 2015 [15]	Phase II	62	Operable stage II–III T1c, cN1-3 or T2-4, any N (all M0) HER2−	*nab*-P 100 mg/m^2^ + carbo AUC 2 qw + vorinostat 400 mg qd on days 1–3 of each week	No invasive cancer in breast and axilla	25.8		NR
				*nab*-P 100 mg/m^2^ + carbo AUC 2 qw + placebo		29.0		
Somlo, 2015 [60]	Phase II	49	Stage II-III LABC or IBC	*nab*-P 100 mg/m^2^ d1, 8, 15, and 22 + carbo AUC 6 d1 (4 × 28-day cycles)	pCR; RCB 0 + 1	53; 65	NR	NA
Mrozek, 2014 [61]	Phase II	33	Stage II–III HER2−	*nab*-P 100 mg/m^2^ + carbo AUC 2 qw 3/4 + bev q2w 10 mg/kg	ypT0 ypN0	18	TNBC: 50	NA
Matsuda, 2015 [62]	Phase II	25	HER2− IBC	Panitumumab 2.5 mg/kg + *nab*-P 100 mg/m^2^ + carbo AUC 2 → FEC	RCB 0	33	HR+: 38 TNBC: 62	NA
Shimada, 2015 [20]	Institutional	53	Stage II–III HER2−	*nab*-P 260 mg/m^2^ q3w → by EC	ypT0/is ypN_any_	5.7	HR+: 2.9 TNBC: 10.5	NA
HER2+ (n = 3)								
Sinclair, 2013 [21]	Phase II	60	Stage II–III	Carbo + *nab*-P 100 mg/m^2^ qw + trastuz qw (4 mg/kg loading, then 2 mg/kg/wk)	No invasive cancer in breast and axilla	45	ER+: 40 ER−: 52	NA
Tanaka, 2015 [22]	Phase II	46	Stage I–III	Anthracycline → *nab*-P 260 mg/m^2^ + trastuz q3w	ypT0/is ypN0	49	ER+: 36 ER−: 71	NA
Zelnak, 2015 [23]	Phase II	27	Stage I–III	*nab*-P 260 mg/m^2^ q2w → vinorelbine 25 mg/m^2^ qw + trastuz qw (4 mg/kg loading, then 2 mg/kg/wk)	No invasive cancer in breast and axilla	48.1	ER/PR+: 18.2 ER/PR−: 68.8	NA

*1c* tumor >10 mm but ≤20 mm, *AC* doxorubicin + cyclophosphamide, *AUC* area under the curve, *BC* breast cancer, *bev* bevacizumab, carbo, carboplatin, *ddAC* dose-dense AC, *EC* epirubicin + cyclophosphamide, *ER* estrogen receptor, *FEC* fluorouracil, epirubicin, and cyclophosphamide, *FE100C* FEC with epirubicin at 100 mg/m^2^, *gem* gemcitabine, *HER2* human epidermal growth factor receptor 2, *HR* hormone receptor, *IBC* inflammatory breast cancer, *ITT* intention to treat, *LABC* locally advanced breast cancer, *M* distant metastasis, *N* regional lymph nodes, *NA* not applicable, *nab*-P *nab*-paclitaxel, *NR* not reported, *pac* paclitaxel, *pCR* pathological complete response, *PEG-G* pegfilgrastim, *qd* once daily, *qw* weekly, *qw 2/3* first 2 of 3 weeks, *qw 3/4* first 3 of 4 weeks, *q2w* every 2 weeks, *q3w* every 3 weeks, *RCB* residual cancer burden, *T* primary tumor, *TNBC* triple-negative breast cancer, *trastuz* trastuzumab, *yp* postneoadjuvant therapy

**Table 2:** Progression-free and overall survival in recent clinical studies of *nab*-paclitaxel in metastatic breast cancer

Study, author, year	Type of study	N (ITT)	Patient population or stage of disease	Line of therapy	Regimen	PFS, months, median	OS, months, median
Unselected (all subtypes; n = 5)							
CALGB 40502, Rugo, 2015 [30]	Phase III	799	Stage IIIC or IV locally recurrent or MBC	First	Bev^a^ + paclitaxel 90 mg/m^2^ qw 3/4	11	27.4^b^
					Bev + *nab*-P 150 mg/m^2^ qw 3/4	9.3	23.5
					Bev + ixabepilone 16 mg/m^2^ qw 3/4	7.4	23.6
Jain, 2016 [32]	Phase II/III	180	MBC	Multiple (all lines)	*nab*-P 260 mg/m^2^ q3w	7.8	NR
					PICN 260 mg/m^2^ q3w	5.3	NR
					PICN 295 mg/m^2^ q3w	8.1	NR
Sun, 2014 [31]	Phase II	73	MBC	Multiple (all lines)	*nab*-P 125 mg/m^2^ qw 3/4 → cisplatin 75 mg/m^2^ q4w	9.8	26.9
Dent, 2013 [63]	Retrospective	43	MBC	Multiple (all lines)	*nab*-P 260 mg/m^2^ q3w	NR	10.8
					*nab*-P 100 mg/m^2^ qw 3/4		13.6
Aigner, 2013 [64]	Retrospective	36	MBC	Multiple (all lines)	*nab*-P 100-150 mg/m^2^ qw	7.5	14.2
HER2− or TNBC (n = 4)							
TBCRC 019, Forero-Torres, 2015 [37]	Phase II	64	Metastatic TNBC	Multiple (all lines)	*nab*-P 100 mg/m^2^ qw 3/4 + tigatuzumab (10 mg/kg, then 5 mg/kg q2w)	2.8	NR
					*nab*-P 100 mg/m^2^ qw 3/4	3.7	
Palumbo, 2015 [38]	Phase II	52	HER2− MBC	Second	*nab*-P 260 mg/m^2^ q3w	8.9	Not yet reached
Hamilton, 2013 [65]	Phase II	34	Metastatic TNBC	First	*nab*-P 100 mg/m^2^ qw 3/4 + carbo AUC 2 qw 3/4 + bev 10 mg/kg q2w	9.2	NR

No HER2+ studies reported OS or PFS

*AUC* area under the curve, *bev* bevacizumab, *carbo* carboplatin, *HER2* human epidermal growth factor receptor 2, *ITT* intention to treat, *MBC* metastatic breast cancer, *nab*-P *nab*-paclitaxel, *NR* not reported, *OS* overall survival, *PFS* progression-free survival, *PICN* paclitaxel injection concentrate for nanodispersion, *qw* weekly, *qw 3/4* first 3 of 4 weeks, *q2w* every 2 weeks, *q3w* every 3 weeks, *q4w* every 4 weeks, *TNBC* triple-negative breast cancer

^a^Bev was optional per protocol amendment; 97% of patients received bev

^b^Median OS was 26.5 months for comparison vs *nab*-P

### Studies of *nab*-paclitaxel in early-stage breast cancer

#### Unselected (all subtypes)

Among 7 phase II and III studies of neoadjuvant *nab*-paclitaxel (majority administered weekly) that did not select for specific disease subtype, the pathological complete response (pCR) rate ranged from 22 to 40%; 4 phase II studies included <50 patients (Table 1).

A trend toward benefit for stage II disease was observed with *nab*-paclitaxel 125 mg/m^2^ once weekly (qw) plus carboplatin vs paclitaxel 80 mg/m^2^ qw plus carboplatin in a phase II trial with a pCR rate of 36.8 vs 15.8% (odds ratio [OR], 3.11; 95% CI 0.963–10.053; *P* = 0.051); however, no such trend was observed in the overall population [9]. More grade 4 neutropenia was observed with *nab*-paclitaxel than with paclitaxel (56.7 vs 21.1%; *P* < 0.001).

A phase II trial of *nab*-paclitaxel 260 mg/m^2^ q3w and cyclophosphamide 600 mg/m^2^ followed by fluorouracil, epirubicin, and cyclophosphamide (FEC) q3w for operable breast cancer resulted in a pCR rate of 37% (95% CI 24–50%) [10]. Hormone receptor–positive/HER2-negative tumors demonstrated the lowest pCR rate (8%), whereas all other molecular subgroups had pCR rates ranging from 56 to 63%. Hormone receptor negativity (HR, 11.9; 95% CI 2.8–52.6; *P* = 0.001) and HER2 positivity (HR, 6.8; 95% CI 1.5–32.0; *P* = 0.015) were independent predictors of pCR.

The large phase III GeparSepto trial compared neoadjuvant paclitaxel 80 mg/m^2^ qw vs *nab*-paclitaxel 125 mg/m^2^ (150 mg/m^2^ before amendment) qw followed by epirubicin and cyclophosphamide (EC) for early-stage breast cancer, with trastuzumab plus pertuzumab added for HER2-positive cancers [11]. The original dose of *nab*-paclitaxel (150 mg/m^2^) was amended to 125 mg/m^2^ due to the frequency of treatment discontinuations and sensory neuropathy. Overall, patients achieved a significantly higher pCR rate with *nab*-paclitaxel vs paclitaxel (38.4 vs 29.0%; *P* < 0.001). The higher pCR rate for *nab*-paclitaxel vs paclitaxel was maintained in the set of patients who received treatment after the *nab*-paclitaxel dose amendment (41.4 vs 32.4%; *P* = 0.013). The largest difference between treatment arms was observed in the triple-negative breast cancer (TNBC) subgroup, with *nab*-paclitaxel achieving a pCR rate of 48.2 vs 26.3% with paclitaxel (*P* < 0.001).

The pCR rates in 4 phase II neoadjuvant studies of *nab*-paclitaxel for the treatment of early-stage breast cancer of unselected subtype ranged from 22 to 40%, with 71 to 77.5% of patients having breast-conserving surgery [9–12].

#### HER2-negative or TNBC

Nine studies of *nab*-paclitaxel in early-stage HER2-negative breast cancer or TNBC were retrieved. The overall pCR rate ranged from 5.7 to 53% (Table 1). Most of these were combination studies.

The phase II Nabrax GEICAM study of neoadjuvant *nab*-paclitaxel 150 mg/m^2^ the first 3 of 4 weeks (qw 3/4) in patients with ER-positive, HER2-negative breast cancer demonstrated an ORR of 76.5% [13]. A residual cancer burden (RCB) score of 0 + 1 was achieved by 24.7% of the treated population (n = 81), and the rate of conversion to breast-conserving surgery was 40%.

The phase II SWOG S0800 trial evaluated the backbone neoadjuvant regimen of *nab*-paclitaxel 100 mg/m^2^ qw with dose-dense doxorubicin plus cyclophosphamide (AC) ± bevacizumab for the treatment of HER2-negative locally advanced or inflammatory breast cancer [14]. The overall pCR rate was 28%, with a significantly higher pCR rate reported in the bevacizumab vs non-bevacizumab arm (36 vs 21%; *P* = 0.021). In hormone receptor–positive disease, the difference in pCR rate between bevacizumab and no bevacizumab was not significant (25 vs 18%; *P* = 0.41). However, patients with hormone receptor–negative tumors demonstrated a significantly improved pCR rate with bevacizumab (59 vs 28%; *P* = 0.014). In addition, a significantly improved pCR rate was achieved with bevacizumab vs no bevacizumab in the locally advanced breast cancer group (37 vs 22%; *P* = 0.035).

Another trial that evaluated a *nab*-paclitaxel–containing backbone regimen was the phase II TBCRC 008 study, which compared 12 weeks of neoadjuvant carboplatin, *nab*-paclitaxel 100 mg/m^2^ qw, and vorinostat vs carboplatin, *nab*-paclitaxel 100 mg/m^2^ qw, and placebo for operable, stage II–III, HER2-negative breast cancer [15]. Similar pCR rates were reported for both arms (vorinostat, 25.8% vs placebo, 29.0%). The pCR rates in patients with TNBC were 41.7% with vorinostat and 58.3% with placebo.

The phase II ADAPT trial was designed as an umbrella trial in which patients with early-stage breast cancer had 2 sequential core biopsies during neoadjuvant therapy (baseline and 3 weeks after treatment initiation) to assess early biomarker changes and guide adjuvant therapy selection [16]. Patients were assigned to 1 of 4 subtrials based on molecular subtyping. One subtrial was the ADAPT-TN trial, which evaluated a backbone of neoadjuvant *nab*-paclitaxel 125 mg/m^2^ given the first 2 of 3 weeks (qw 2/3) with either carboplatin or gemcitabine in patients with TNBC [17, 18]. The pCR rate differed significantly between arms (carboplatin, 47.4% vs gemcitabine, 29.7%; *P* = 0.0045). Patients who received *nab*-paclitaxel plus gemcitabine vs *nab*-paclitaxel plus carboplatin experienced a significantly higher frequency of dose reductions (20.6 vs 11.9%; *P* = 0.03), treatment-related severe adverse events (13 vs 5%; *P* = 0.02), grade 3–4 infections (6.1 vs 1.3%; *P* = 0.04), and alanine aminotransferase elevations (11.7 vs 3.3%; *P* = 0.01).

Docetaxel 75 mg/m^2^ q3w was compared with *nab*-paclitaxel 100 mg/m^2^ qw 3/4 followed by FEC with epirubicin at 100 mg/m^2^ for stage II–III, HER2-negative breast cancer [19]. The overall pCR rate was 17%, with a higher pCR rate of 30% in patients with TNBC. Another trial that evaluated sequential *nab*-paclitaxel 260 mg/m^2^ q3w and EC demonstrated a pCR rate of 5.7% in patients with stage II–III HER2-negative breast cancer (N = 53) [20].

#### HER2-positive

Three studies of *nab*-paclitaxel in early-stage HER2-positive breast cancer demonstrated highly consistent pCR rates, ranging from 45 to 49% (Table 1). Neoadjuvant carboplatin, *nab*-paclitaxel, and trastuzumab treatment in patients with stage II–III HER2-positive tumors resulted in a pCR rate of 45% in 55 evaluable patients, and a pCR plus RCB I rate of 50% in the ITT population [21]. A pCR rate of 52% was achieved in patients with ER-negative disease compared with a pCR rate of 40% in those with ER-positive disease. A phase II trial of neoadjuvant anthracycline followed by *nab*-paclitaxel 260 mg/m^2^ q3w plus trastuzumab for operable HER2-positive breast cancer reported a pCR rate of 49% in the treated population [22]. Subgroup analysis demonstrated a higher pCR rate of 71% in patients with ER-negative tumors vs 36% in those with ER-positive disease. Similar results were achieved with neoadjuvant *nab*-paclitaxel 260 mg/m^2^ every 2 weeks (q2w) followed by vinorelbine and trastuzumab for stage I–III HER2-positive breast cancer [23]. An overall pCR rate of 48.1% was reported, with a pCR rate of 68.8% in the hormone receptor–negative population and a pCR rate of 18.2% in the hormone receptor–positive population (n = 11).

#### Post hoc analyses

Recent post hoc biomarker analyses were reported for the Brown University Oncology Group trials BR-211A (NCT00723125), which evaluated bevacizumab, *nab*-paclitaxel, and carboplatin in stage II–III HER2-negative breast cancer, and BR-211B (NCT00617942), which examined trastuzumab, *nab*-paclitaxel, and carboplatin in stage II–III HER2-positive breast cancer [24–26]. Among patients with HER2-negative early-stage breast cancer in BR-211A, a strong correlation was found between triple-negative status and pCR rate after treatment with bevacizumab, *nab*-paclitaxel, and carboplatin (*P* < 0.001) [25]. In the BR-211B trial, the pCR rate was 50% among 20 patients for whom evaluable tissues and pCR data were available, and a strong correlation was reported for high baseline HER2 and pCR (*P* = 0.002) [24]. In addition, higher baseline levels of stromal tumor-infiltrating lymphocytes (sTILs) (median, 35 vs 25%; *P* = 0.018) were found in patients with HER2-positive tumors who achieved RCB 0 + 1 (defined as responders) vs RCB 2 + 3 (defined as nonresponders), respectively [26].

#### Adjuvant treatment with nab-paclitaxel

Two studies of adjuvant *nab*-paclitaxel in early-stage disease were retrieved. The combination of *nab*-paclitaxel 100 mg/m^2^ and capecitabine was compared with EC or cyclophosphamide, methotrexate, and fluorouracil (CMF) as adjuvant therapy for early-stage breast cancer in nonfrail elderly patients (age ≥65 years) in the phase II ICE II-GBG 52 trial [27]. After a median follow-up of almost 23 months, no significant difference in OS was observed between treatment arms (HR, 1.18; 95% CI 0.52–2.66). A greater percentage of patients experienced treatment discontinuations with *nab*-paclitaxel plus capecitabine than EC/CMF (35.8 vs 6.6%; *P* < 0.001). In both arms, the main reasons for discontinuation were adverse events followed by investigator or patient decision; however, grade ≥3 toxicities were less frequent with *nab*-paclitaxel plus capecitabine than EC/CMF (64.8 vs 90.9%; *P* < 0.001). Grade ≥3 hematologic events were less common (22.3 vs 88.4%; *P* < 0.001) and grade ≥3 nonhematologic events were more common (58.5 vs 18.7%; *P* < 0.001) with *nab*-paclitaxel plus capecitabine than with EC/CMF. The authors suggested that tolerability might have been better if a lower capecitabine dose were used in the *nab*-paclitaxel plus capecitabine arm.

#### Ongoing trials in early-stage breast cancer

The ongoing phase III GAIN-2 study (NCT01690702; planned N = 2886) compares *nab*-paclitaxel on a less common dose-dense schedule (330 mg/m^2^ q2w) plus EC vs EC followed by docetaxel using invasive disease-free survival as the primary endpoint (https://clinicaltrials.gov/ct2/show/NCT01690702, [28]). Among the 1473 patients who have been randomized, those in the *nab*-paclitaxel arm demonstrated higher rates of grade ≥3 febrile neutropenia (12 vs 8%) and peripheral sensory neuropathy (83 vs 68%) and required more dose reductions due to hematologic toxicities (30 vs 10%; *P* < 0.001).

The ETNA trial, another ongoing phase III study (NCT01822314; planned N = 632), is evaluating single-agent *nab*-paclitaxel 125 mg/m^2^ qw 3/4 vs paclitaxel 90 mg/m^2^ qw 3/4 as neoadjuvant therapy for high-risk HER2-negative breast cancer [https://clinicaltrials.gov/ct2/show/NCT01822314]. In each arm, patients will receive AC, EC, or FEC after initial taxane therapy. The primary endpoint is pCR.

Another ongoing phase III trial (Nordic trip, N = 1800), will provide additional information about the potential benefit of *nab*-paclitaxel plus EC as a treatment for early TNBC [29]. Patients will be randomized to receive adjuvant or neoadjuvant treatment in 1 of 3 arms: *nab*-paclitaxel followed by EC, *nab*-paclitaxel plus capecitabine followed by EC plus capecitabine, or *nab*-paclitaxel plus carboplatin followed by EC. The primary endpoint is invasive disease-free survival.

### Studies of *nab*-paclitaxel in MBC

#### Unselected (all subtypes)

Four studies of *nab*-paclitaxel in MBC of unselected subtype reported median OS ranging from 10.8 months with *nab*-paclitaxel 260 mg/m^2^ q3w to 26.9 months with *nab*-paclitaxel 125 mg/m^2^ qw 3/4 combined with cisplatin (Table 2).

The phase III CALGB 40502 trial evaluated first-line bevacizumab combined with paclitaxel 90 mg/m^2^, *nab*-paclitaxel 150 mg/m^2^, or ixabepilone 16 mg/m^2^ qw 3/4 for locally recurrent or metastatic breast cancer [30]. A protocol amendment made the use of bevacizumab optional; however, 97% of patients had already received bevacizumab at that time. The majority (93%) of patients had HER2-negative disease. Median PFS (primary endpoint) was 11 months for the paclitaxel arm vs 9.3 months with *nab*-paclitaxel (HR, 1.20; 95% CI 1.00–1.45; *P* = 0.054) and 7.4 months with ixabepilone (HR, 1.59; 95% CI 1.31–1.93; *P* < 0.001). Median OS significantly differed between ixabepilone and paclitaxel (23.6 vs 27.4 months, respectively; HR, 1.31; 95% CI 1.03–1.66; *P* = 0.027), but not between *nab*-paclitaxel and paclitaxel (23.5 vs 26.5 months, respectively; HR, 1.17; 95% CI 0.92–1.47; *P* = 0.20). Grade ≥3 nonhematologic toxicities were more common in the *nab*-paclitaxel vs paclitaxel arm (65 vs 49%; *P* < 0.001), with grade ≥2 sensory neuropathy affecting more patients treated with *nab*-paclitaxel vs paclitaxel (54 vs 46%; *P* = 0.031). Compared with paclitaxel, *nab*-paclitaxel demonstrated worse hematologic and nonhematologic toxicity (both, *P* < 0.001). Ixabepilone caused less hematologic toxicity (*P* = 0.004) but not significantly different nonhematologic toxicity (*P* = 0.14) than paclitaxel. Dose reductions occurred more frequently and earlier in the *nab*-paclitaxel arm: by cycle 3, 45% of patients receiving *nab*-paclitaxel had undergone a dose reduction compared with 15% of those receiving paclitaxel and 15% of those receiving ixabepilone. Only 28% of patients received full-dose *nab*-paclitaxel at cycle 5 vs 76% of patients receiving paclitaxel and 65% receiving ixabepilone.

A phase II trial of *nab*-paclitaxel 125 mg/m^2^ qw 3/4 and cisplatin for MBC reported an ORR (primary endpoint) of 67.1%, with an 80.6% ORR in the first-line setting and an 80% ORR for those who were not previously treated with taxanes [31]. The median PFS was 9.8 months (95% CI 8.1–11.6 months) by investigator assessment, and median OS was 26.9 months. Compared with patients who were pretreated with taxanes, those who had not previously received taxanes demonstrated longer PFS (median, 8.5 vs 11.2 months; *P* = 0.009) by investigator assessment and longer OS (median, not reached vs 16.7 months; *P* < 0.001). There were no significant differences in PFS according to molecular subtype. Treatment was well tolerated in most patients, with neutropenia being the most common cause for dose adjustment. Grade ≥3 neutropenia was the most common hematologic adverse event, affecting 84.9% of patients.

A randomized phase II/III trial of women with refractory MBC compared *nab*-paclitaxel 260 mg/m^2^ q3w with paclitaxel concentrate for nanodispersion (PICN) 260 or 295 mg/m^2^ q3w [32]. Comparing the equal-dose regimens of *nab*-paclitaxel and PICN, ORR and PFS were numerically greater for *nab*-paclitaxel vs PICN (ORR, 43 vs 35%; PFS, median, 7.8 vs 5.3 months [*P* not significant for either]). ORR and PFS were not significantly different for higher-dose PICN vs lower-dose PICN or vs *nab*-paclitaxel. The lower-dose PICN arm had lower rates of grade ≥3 adverse events compared with the higher-dose PICN arm and the *nab*-paclitaxel arm.

A number of regional retrospective analyses have also been conducted recently on the use of *nab*-paclitaxel for the treatment of unselected MBC. A retrospective analysis of patients with breast cancer in British Columbia who received *nab*-paclitaxel from 2007 to 2011 was performed [33]. Most patients had metastatic disease, and 2 had regional relapse only. Approximately one-fourth of patients had prior taxane exposure. Time to relapse was significantly shorter in patients with prior exposure to adjuvant taxanes vs those without (median, 2.7 vs 4.5 years, *P* < 0.001). No significant differences in time to treatment failure (defined as time from first to last cycle of *nab*-paclitaxel) or dose reduction rates were found between these 2 groups. Thus, *nab*-paclitaxel may result in clinical benefit in patients with MBC regardless of whether they have had prior taxane exposure. A retrospective German survey of national chemotherapy practices demonstrated that *nab*-paclitaxel was used less frequently than paclitaxel and docetaxel for first-line treatment of MBC [34]. However, retrospective analysis of a US claims database of patients with MBC who were treated with taxanes (n = 2599 docetaxel; n = 1643 paclitaxel; n = 261 *nab*-paclitaxel) demonstrated that patients remained on *nab*-paclitaxel 50% longer than on other taxanes (127 vs 85 days; *P* < 0.05), possibly due to lower incidence of discontinuation for neutropenia with *nab*-paclitaxel vs docetaxel or paclitaxel (6.9% vs docetaxel, 29.4% or paclitaxel, 17.5%; *P* < 0.001) [35]. A separate analysis of a US claims database revealed that *nab*-paclitaxel was most often administered as second- or later-line therapy, as monotherapy, or on a weekly schedule [36].

#### HER2-negative or TNBC

A phase II trial of *nab*-paclitaxel 100 mg/m^2^ qw 3/4 with or without anti–death receptor 5 monoclonal antibody tigatuzumab (10 mg/kg loading, 5 mg/kg q2w) was performed in patients with metastatic TNBC [37]. ORR was 28% in the combination arm vs 38% in the *nab*-paclitaxel–alone arm. PFS was not significantly different between arms (median, 3.7 months for *nab*-paclitaxel monotherapy vs 2.8 months for the combination; *P* = 0.3152). Five patients in the combination arm demonstrated long-term PFS (334–1025+ days) compared with 1 patient in the *nab*-paclitaxel arm (1004+ days).

There was one study of *nab*-paclitaxel in patients with HER2-negative MBC (unselected for hormone receptor status) that reported median PFS [38]. None of the studies on HER2-negative breast cancer or TNBC reported OS.

A prospective trial of second-line *nab*-paclitaxel 260 mg/m^2^ q3w in patients with HER2-negative MBC with prior taxane exposure reported an ORR of 48% (95% CI 31.5–61.3%) and a median PFS of 8.9 months (95% CI 8.0–11.6 months; range, 5–21+ months) [38]. Response rate by subgroup demonstrated a higher response in TNBC (68.8%) vs other subgroups (ER+/PR−, 55.6%; ER−/PR+, 50.0%; ER+/PR+, 32.0%). Median OS was not reached.

#### Health economic analyses

Three cost-effectiveness analyses compared *nab*-paclitaxel with other taxanes in patients with MBC.

The Spanish COSTABRAX cost-effectiveness analysis demonstrated that *nab*-paclitaxel q3w was cost-effective compared with paclitaxel q3w as a second-line treatment for MBC [39]. Efficacy data from the phase III CA012 trial were used in a Markov model expanded to a time horizon of 5 years. The cost of life-year gained with *nab*-paclitaxel q3w vs paclitaxel q3w was €11,088, and the cost per quality-adjusted life-year (QALY) gained was €17,808. Compared with paclitaxel qw, *nab*-paclitaxel q3w showed a savings of €711 per patient. Another study combined cost data in China with a meta-analysis of 10 randomized phase III trials of *nab*-paclitaxel 260 mg/m^2^ q3w or docetaxel 100 mg/m^2^ q3w in comparison with paclitaxel 175 mg/m^2^ q3w [40]. The cost per course of treatment was $19,752 for *nab*-paclitaxel, $8940 for paclitaxel, and $13,741 for docetaxel. The cost per QALY gained for *nab*-paclitaxel vs docetaxel as alternatives to paclitaxel was $57,900 vs $130,600. Thus, *nab*-paclitaxel appeared to be a more cost-effective alternative to docetaxel as initial therapy for MBC in a Chinese healthcare setting. The Italian COSTANza study, which used a Markov model, also suggested that *nab*-paclitaxel was cost-effective, with a gain of 0.165 QALY compared with paclitaxel [41].

A recent questionnaire-based study of healthcare providers (N = 22) in Sweden indicated that mean infusion times for *nab*-paclitaxel vs paclitaxel were 42.1 ± 20.7 vs 104.3 ± 43.3 min, respectively [42]. Total patient times in clinic per infusion were 82.2 ± 40.9 and 183.9 ± 34.8 min, respectively. The study suggested that the corresponding time required for a 12-week treatment of *nab*-paclitaxel q3w, *nab*-paclitaxel qw, and paclitaxel qw would be 2.8, 8.4, and 20.9 h, respectively. The corresponding time for patient hospital visits would be 5.5, 16.4, and 36.8 h, respectively. Thus, *nab*-paclitaxel may require less time for drug administration, potentially reducing cost.

#### Ongoing trials in MBC

Table 3 lists selected ongoing trials of *nab*-paclitaxel regimens for the treatment of MBC. There are several ongoing trials evaluating combinations of *nab*-paclitaxel with HER2-targeted agents for the treatment of metastatic HER2-positive breast cancer. The single-arm phase III PERUSE trial (NCT01572038; planned N = 1500) is evaluating the safety of first-line pertuzumab combined with trastuzumab and a taxane of the investigator’s choice, as its primary endpoint, in patients with metastatic or locally recurrent HER2-positive breast cancer [https://clinicaltrials.gov/ct2/show/NCT01572038]. Secondary endpoints include PFS, OS, ORR, and quality of life. Interim safety results indicated that grade ≥3 adverse events occurred in 53.4% of patients in the docetaxel group (n = 320), 41.1% in the paclitaxel group (n = 331), and 26.7% in the *nab*-paclitaxel group (n = 45) [43]. The most common grade ≥3 toxicities included neutropenia (approximately 12, 6, and 2% in docetaxel, paclitaxel, and *nab*-paclitaxel groups, respectively) and diarrhea (approximately 9, 6, and 6%). The phase IIIb SAPPHIRE trial (NCT02019277; N = 50) is evaluating the safety and efficacy of trastuzumab combined with intravenous pertuzumab and a taxane of investigator’s choice in patients with metastatic HER2-positive breast cancer [44, 45]. Interim results showed that 50 patients had been enrolled, with the majority (72%) receiving *nab*-paclitaxel as the taxane of choice. Grade ≥3 adverse events were reported in 52% of patients, although toxicities were not categorized according to type of taxane received. In addition, the phase I/II STELA trial (NCT02073916; planned N = 45) will combine trastuzumab emtansine (T-DM1), *nab*-paclitaxel, and lapatinib to treat metastatic HER2-positive MBC (https://clinicaltrials.gov/ct2/show/NCT02073916, [46]).

**Table 3:** Selected ongoing studies of *nab*-paclitaxel in all stages of breast cancer

Study, ClinicalTrials.gov identifier	Phase	Planned N	Patient population or stage of disease	Regimen	Primary endpoint
Early-stage (n = 4)					
GAIN-2, NCT01690702 [66]	III	2886	High risk, after R0 resection	Adjuvant epirubicin 150 mg/m^2^ q2w × 3 cycles → *nab*-P 260-330 mg/m^2^ × 3 cycles (TBD in run-in-phase) q2w → cyclophosphamide 2000 mg/m^2^ q2w × 4 cycles	iDFS
				EC q2w → docetaxel q2w	
ETNA, NCT01822314	III	632	High risk HER2−	Neoadjuvant *nab*-P 125 mg/m^2^ qw 3/4 × 4 cycles → AC, EC, or FEC × 4 cycles	pCR
				Neoadjuvant paclitaxel 90 mg/m^2^ qw 3/4 × 4 cycles → AC, EC, or FEC × 4 cycles	
NCT00618657	II	120	Stage I–III	Neoadjuvant *nab*-P + carbo + trastuz for HER2+ qw × 12 weeks	PFS
				Neoadjuvant *nab*-P + carbo qw × 12 cycles + bev q2w × 5 cycles for HER2−	
NCT02530489	II	37	TNBC nonmetastatic	Neoadjuvant *nab*-P 100 mg/m^2^ + atezolizumab	pCR
NCT02489448	I/II	61	Stage I–III TNBC	Neoadjuvant durvalumab + *nab*-P qw × 12 cycles → ddAC × 4 cycles	pCR (ypT0/Tis, ypN0)
Metastatic or advanced stage (n = 12)					
PERUSE, NCT01572038 [43]	III	1500	HER2+	Trastuz + pertuzumab + taxane of choice	Safety
IMpassion130, NCT02425891	III	350	Untreated locally advanced or metastatic TNBC	*nab*-P + atezolizumab	PFS
				*nab*-P + placebo	
tnAcity, NCT01881230 [48]	II/III	790	TNBC	Selected *nab*-P regimen from phase II portion (either *nab*-P 125 mg/m^2^ + gem 1000 mg/m^2^ d1, 8 q3w or *nab*-P 125 mg/m^2^ + carbo AUC 2 d1, 8 q3w)	PFS
				Gem 1000 mg/m^2^ + carbo AUC 2 d1, 8 q3w	
SNAP, NCT01746225 [48]	II	258	HER2− MBC	Induction *nab*-P 125 mg/m^2^ qw 3/4 × 3 cycles in all patients → randomization into 3 arms: *nab*-P 150 mg/m^2^ q2w	ORR by RECIST v1.1
				*nab*-P 100 mg/m^2^ qw 3/4	
				*nab*-P 75 mg/m^2^ qw	
NCT00733408	II	63	MBC	Induction *nab*-P qw 3/4 + bev q2w → maintenance with bev q2w or q3w + erlotinib qd	PFS
NCT01730833	II	50	Stage II–IV HER2+ LABC and MBC	Pertuzumab q3w + trastuz qw + *nab*-P qw	PFS
NCT01463072	II	40	LABC or MBC in ≥65-year-old patients	*nab*-P qw 3/4	Tolerability
PembroPlus, NCT02331251	I/II	90	MBC and other solid tumor types	Pembrolizumab + chemotherapy, including *nab*-P	RP2D
NCT02379247	I/II	54	Locally recurrent BC or MBC	PI3K inhibitor BYL719 + *nab*-P 100 mg/m^2^ qw 3/4	Phase I, RP2D; phase II, ORR
NCT01938833	I/II	47	Metastatic inflammatory BC	*nab*-P + romidepsin qw 3/4	MTD, PFS
STELA, NCT02073916 [46]	I/II	45	HER2+ MBC	T-DM1 + lapatinib + *nab*-P	MTD
NCT02309177	I	138^a^	Recurrent MBC and other solid tumor types	Nivolumab + *nab*-P 100 mg/m^2^ qw 3/4^a^	DLTs, safety
				Nivolumab + *nab*-P 260 mg/m^2^ q3w^a^	

*AC* doxorubicin + cyclophosphamide, *AUC* area under the curve, *BC* breast cancer, *bev* bevacizumab, *carbo* carboplatin, *ddAC* dose-dense AC, *DLT* dose-limiting toxicity, *EC* epirubicin + cyclophosphamide, *FEC* fluorouracil, epirubicin, and cyclophosphamide, *gem* gemcitabine, *HER2* human epidermal growth factor receptor 2, *iDFS* invasive disease-free survival, *is* in situ, *LABC* locally advanced breast cancer, *MBC* metastatic breast cancer, *MTD* maximum tolerated dose, *nab*-P *nab*-paclitaxel, *ORR* overall response rate, *pCR* pathologic complete response, *PD-L1* programmed death-ligand 1, *PFS* progression-free survival, *PI3K* phosphoinositide 3-kinase, *q2w* every 2 weeks, *q3w* every 3 weeks, *qd* daily, *qw* weekly, *qw* 3*/4* first 3 of 4 weeks, *R* resection margin, *RECIST* Response Evaluation Criteria In Solid Tumors, *RP2D* recommended phase 2 dose, *T* primary tumor, *TBD* to be determined, *T-DM1* trastuzumab emtansine, *TNBC* triple-negative breast cancer, *trastuz* trastuzumab, *yp* postneoadjuvant therapy

^a^Pertains to MBC arms only

A number of ongoing trials are also evaluating *nab*-paclitaxel in HER2-negative MBC. The phase II/III tnAcity trial (NCT01881230) is comparing *nab*-paclitaxel plus gemcitabine with *nab*-paclitaxel plus carboplatin as first-line treatment for metastatic TNBC. The phase II portion (N = 240) has 3 arms: *nab*-paclitaxel 125 mg/m^2^ plus gemcitabine 1000 mg/m^2^ on days 1 and 8 q3w, *nab*-paclitaxel 125 mg/m^2^ plus carboplatin area under the curve (AUC) of 2 days 1 and 8 q3w, and gemcitabine 1000 mg/m^2^ plus carboplatin AUC of 2 days 1 and 8 q3w (https://clinicaltrials.gov/ct2/show/NCT01881230, [47]). In the phase III portion (N = 550), the *nab*-paclitaxel plus gemcitabine or *nab*-paclitaxel plus carboplatin arm will be selected based on phase II trial results and compared with gemcitabine 1000 mg/m^2^ plus carboplatin AUC of 2 q3w. The phase II SNAP trial (NCT01746225; planned N = 258) will evaluate different schedules of first-line *nab*-paclitaxel for the treatment of HER2-negative MBC (https://clinicaltrials.gov/ct2/show/NCT01746225, [48]). All patients will receive induction *nab*-paclitaxel 125 mg/m^2^ qw 3/4 followed by *nab*-paclitaxel 150 mg/m^2^ on days 1 and 15 of a 28-day cycle, 100 mg/m^2^ qw 3/4, or 75 mg/m^2^ qw. PFS will be assessed as the primary endpoint. An ongoing phase I/II study (NCT01938833; planned N = 47) is evaluating the combination of *nab*-paclitaxel plus the histone deacetylase inhibitor romidepsin in recurrent or metastatic HER2-negative inflammatory breast cancer [https://clinicaltrials.gov/ct2/show/NCT01938833]. Results from the phase I portion (n = 9) demonstrated that the regimen was well tolerated and resulted in an ORR of 33%, including 1 complete response [49].

## Discussion

Recent clinical data indicate that *nab*-paclitaxel is effective and safe across all stages of breast cancer. The results from trials in the neoadjuvant setting for early-stage TNBC or HER2-positive breast cancer were particularly encouraging. In TNBC, *nab*-paclitaxel monotherapy or in combination with other agents resulted in pCR rates ranging from 10.5 to 62%. In the phase III neoadjuvant GeparSepto trial, the largest difference in pCR was identified for patients with TNBC (*nab*-paclitaxel, 48.2% vs paclitaxel, 26.3%; *P* < 0.001), supporting the clinical benefit of *nab*-paclitaxel in early-stage TNBC [11]. The unmet need for the treatment of TNBC lends greater importance to these findings. Patients with early-stage HER2-positive breast cancer also benefited from *nab*-paclitaxel treatment. Neoadjuvant *nab*-paclitaxel combined with trastuzumab and carboplatin, anthracycline, or vinorelbine demonstrated pCR rates in the breast and lymph nodes ranging from 45 to 49%, which is comparable to those observed for other current neoadjuvant therapies [50]. In addition, neoadjuvant *nab*-paclitaxel resulted in breast-conserving surgery in 71 to 77.5% of patients with early-stage breast cancer.

In the phase III CALGB 40502 study, patients with MBC treated with first-line *nab*-paclitaxel plus bevacizumab achieved a median PFS of approximately 9 months and a median OS of 23.5 months [30]. For reference, patients with MBC who received *nab*-paclitaxel 260 mg/m^2^ q3w as first- or later-line therapy in a phase III trial demonstrated a median time to tumor progression of 5.3 months and a median OS of 15.0 months [5]. Neither PFS nor OS for the *nab*-paclitaxel plus bevacizumab arm of the CALGB 40502 trial was significantly different from that of the paclitaxel plus bevacizumab arm [30]. The 150 mg/m^2^ dose of *nab*-paclitaxel was not optimal, with a higher percentage of patients in the *nab*-paclitaxel group developing hematologic and nonhematologic toxicities. Several ongoing trials are evaluating the potential clinical benefit of *nab*-paclitaxel in patients with MBC, particularly the HER2-positive and TNBC subpopulations. Interim analyses from some of these trials have demonstrated promising results; once final, the findings from these trials will provide further insights into the role of *nab*-paclitaxel for the treatment of breast cancer across treatment settings and patient subsets.

Recent efforts to maintain efficacy while limiting toxicity have focused on the optimization of *nab*-paclitaxel schedule and dose. The safety profiles of *nab*-paclitaxel–based regimens in the recent studies included in this review were consistent with those in past studies, including the registrational phase III trial. The most common grade 3/4 hematologic and nonhematologic adverse events associated with *nab*-paclitaxel were neutropenia and peripheral neuropathy, respectively. The majority of recent studies have examined weekly dosing, likely because an accumulation of data in the metastatic setting suggests an advantage over every-3-week dosing in balancing efficacy and tolerability. In addition, safety and treatment-exposure results of 2 large trials (GeparSepto in the neoadjuvant setting and CALGB 40502 in the metastatic setting) have suggested that *nab*-paclitaxel may be more feasible at a starting dose of 125 mg/m^2^ compared with 150 mg/m^2^ [11, 30].

### Future of *nab*-paclitaxel in breast cancer: *nab*-paclitaxel and immune therapy

In addition to the ongoing trials discussed above, there is interest in combining *nab*-paclitaxel with immuno-oncology agents (Table 3). Chemotherapy-induced cytotoxicity has been shown to activate the immune response and to release tumor antigens from cancer cells [51, 52]. Preclinical data from mouse models of multiple solid tumor types suggested potential synergy between chemotherapy and immune checkpoint inhibitors [53, 54]. Recent clinical data indicated that combining *nab* **-**paclitaxel with checkpoint inhibitors may be safe and effective in MBC. A phase Ib study of atezolizumab, a programmed death ligand 1 (PD-L1) inhibitor, combined with *nab*-paclitaxel qw (NCT01633970) demonstrated activity in 24 efficacy-evaluable patients with metastatic TNBC (ORR 70.8%; stable disease in 20.8%) [55]. Five patients (16%) discontinued *nab*-paclitaxel due to toxicity (3 for peripheral neuropathy [1 each for grades 1, 2, and 3] and 1 each for fatigue and asthenia [both grade 2]). *nab*-Paclitaxel plus atezolizumab is currently being compared with *nab*-paclitaxel plus placebo as a first-line treatment for metastatic TNBC in the randomized phase III IMpassion130 trial (NCT02425891; Table 3) (https://clinicaltrials.gov/ct2/show/NCT02425891, [56]). The PD-1 inhibitor nivolumab is also being evaluated in combination with *nab*-paclitaxel in MBC in an ongoing phase I trial (NCT02309177; planned N = 138) [https://clinicaltrials.gov/ct2/show/NCT02309177]. The combination of atezolizumab and *nab*-paclitaxel is also being evaluated as a neoadjuvant regimen for the treatment of early-stage TNBC in an ongoing phase II trial (NCT02530489; planned N = 37) [https://clinicaltrials.gov/ct2/show/NCT02530489] and a phase III trial (NeoTRIPaPDL1; NCT02620280 [*nab*-paclitaxel plus carboplatin ± atezolizumab]; planned N = 272) [https://clinicaltrials.gov/ct2/show/NCT02620280]. Similarly, the PD-L1 inhibitor durvalumab combined with *nab*-paclitaxel is being examined as neoadjuvant therapy for early-stage TNBC in an ongoing phase I/II trial (NCT02489448; planned N = 61) [https://clinicaltrials.gov/ct2/show/NCT02489448]. Results from these trials will provide further rationale for combining *nab*-paclitaxel with immune therapies as an exciting new treatment approach for early-stage or metastatic breast cancer.

## Conclusions

In addition to demonstrated efficacy in the already established setting of MBC, *nab*-paclitaxel appears to be an effective and well-tolerated neoadjuvant therapy for patients with early-stage breast cancer, particularly the HER2-positive and TNBC subgroups. Ongoing trials are evaluating *nab*-paclitaxel in all stages and subtypes of breast cancer. One anticipated future role of *nab*-paclitaxel is as a backbone chemotherapy, and ongoing trials of *nab*-paclitaxel combined with immune checkpoint inhibitors are particularly exciting, as these may provide more effective treatment regimens for early-stage and metastatic breast cancer.

## Abbreviations

AC: doxorubicin plus cyclophosphamide
ASCO: American Society for Clinical Oncology
AUC: area under the curve
CMF: cyclophosphamide, methotrexate, and fluorouracil
EC: epirubicin and cyclophosphamide
ER: estrogen receptor
FEC: fluorouracil, epirubicin, and cyclophosphamide
HER2: human epidermal growth factor receptor 2
HR: hazard ratio
MBC: metastatic breast cancer
ITT: intention to treat
OR: odds ratio
ORR: overall response rate
OS: overall survival
pCR: pathological complete response
PD-L1: programmed death ligand 1
PFS: progression-free survival
PICN: paclitaxel concentrate for nanodispersion
PRISMA: preferred reporting items for systematic reviews and meta-analyses
QALY: quality-adjusted life-year
q2w: every 2 weeks
q3w: every 3 weeks
qw: once weekly
qw 3/4: first 3 of 4 weeks
RCB: residual cancer burden
sTIL: stromal tumor-infiltrating lymphocytes
T-DM1: trastuzumab emtansine
TNBC: triple-negative breast cancer
